# Lack of evidence of ERVWE1/Xq22.3 involvement in MRSV transcripts detected in Multiple Sclerosis patients

**DOI:** 10.1186/1742-4690-8-S1-A217

**Published:** 2011-06-06

**Authors:** Camila M Romano, Luiz H S Nali, Thiago S Faria, Guilherme S Olival, Jose E Vidal, Maria C D Fink, Laura M Sumita, Augusto C P Oliveira

**Affiliations:** 1 Department of Infectious and Parasitic Diseases, Institute of Tropical Medicine, School of Medicine, University of São Paulo, São Paulo, SP, 05403-000, Brazil; 2 Department of Neurology, Santa Casa de São Paulo, Faculty of Medical Sciences, São Paulo, Brazil; 3 HTLV Service, Institute of Infectious Diseases Emillio Ribas, São Paulo,01246-000, Brazil

## 

HERV are viral ‘fossils’ that constitute 8% of the human genome and have been implicated in both health and disease conditions. HERV-W/MRSV (Multiple Sclerosis associated RetroVirus) RNA has been detected in patients with multiple sclerosis. MS is a chronic inflammatory disease of the central nervous system of unknown cause, resulting in demyelization and axonal degeneration. MSRV is defined by different transcripts, which vary in their source. Recently, it was described that most of MSRV/Env transcripts from MS patients derived from Xq22.3, 15q21.3 and 6q21. Although the genomic Xq22.3 MRSV/Env is truncated due to a stop codon (TGA) at position 39, the transcripts associated harbored a tryptophan (TGG) instead. Thus, to evaluate if a polymorphism at this position could be involved with full expression of this locus in MS patients we sequenced 1085bp of the ERVWE1/Xq22.3 from 15 MS patients of different ethnic origins and different clinical presentations and 14 health individuals. We found that all MS individuals harbor a stop codon at position 39, undermining the expression of a full-length ENV protein. No additional aminoacid substitution was found in both groups. Also, since there is no LTR flanking this gene, we performed an automated search for promoter sequences in 10kb nearby region. We found several putative binding sites for cellular co-factors and enhancers, suggesting that transcription may occur via alternative promoters. Altogether, the findings suggest that transcripts associated to ERVWE1/Xq22.3 previously detected may be artificially generated, possible due to in vitro recombination between this locus and other active ERVW/Env.

